# An Atlas of Genomic Resources for Studying Rosaceae Fruits and Ornamentals

**DOI:** 10.3389/fpls.2021.644881

**Published:** 2021-04-01

**Authors:** Muzi Li, Yuwei Xiao, Steve Mount, Zhongchi Liu

**Affiliations:** Department of Cell Biology and Molecular Genetics, University of Maryland, College Park, MD, United States

**Keywords:** Rosaceae fruits, Rosaceae ornamentals, genome assembly, genome annotation, databases, domestication, origin of species

## Abstract

Rosaceae, a large plant family of more than 3,000 species, consists of many economically important fruit and ornamental crops, including peach, apple, strawberry, raspberry, cherry, and rose. These horticultural crops are not only important economic drivers in many regions of the world, but also major sources of human nutrition. Additionally, due to the diversity of fruit types in Rosaceae, this plant family offers excellent opportunities for investigations into fleshy fruit diversity, evolution, and development. With the development of high-throughput sequencing technologies and computational tools, an increasing number of high-quality genomes and transcriptomes of Rosaceae species have become available and will greatly facilitate Rosaceae research and breeding. This review summarizes major genomic resources and genome research progress in Rosaceae, highlights important databases, and suggests areas for further improvement. The availability of these big data resources will greatly accelerate research progress and enhance the agricultural productivity of Rosaceae.

## Introduction

Rosaceae is a large angiosperm family consisting of three subfamilies—Rosoideae, Amygdaloideae, and Dryadoideae—and ~3,000 species (Xiang et al., 2017). The Rosaceae family of plants is diverse in architecture, including herbs, shrubs, and trees, and has a large number of hybrids and ploidy levels. Most importantly, Rosaceae fruits and ornamentals, such as apple, pear, peach, plum, cherry, almond, strawberry, raspberry, flowering cherry, and rose, are of tremendous economic and agronomic value. Rosaceae fruits are also surprisingly diverse in morphology and fruit type, including fleshy pome, drupe, and achenetum as well as dry fruits (Xiang et al., 2017; Liu Z. et al., 2020). Therefore, the Rosaceae family is also an ideal family for investigations of fruit diversity, domestication, and evolution.

Second- and third-generation sequencing technologies have allowed genome sequencing and genome-wide analyses to revolutionize plant research. The increasing number of sequenced plant genomes and higher quality genomes make molecular research, genome editing, and marker-assisted breeding possible in species previously recalcitrant to molecular genetic research. Further, the establishment of various online databases provides easy access and interaction with the genomic data. These databases help organize genomic resources, facilitate data sharing, and enable genome comparison across different species. In this review, we summarize the latest genome assemblies and annotations of major Rosaceae species, giving examples of findings enabled by genome sequencing. In addition, we present databases useful for the study of Rosaceae species.

## Genome Sequencing and Assemblies of Rosaceae Species

**Figure 1:**
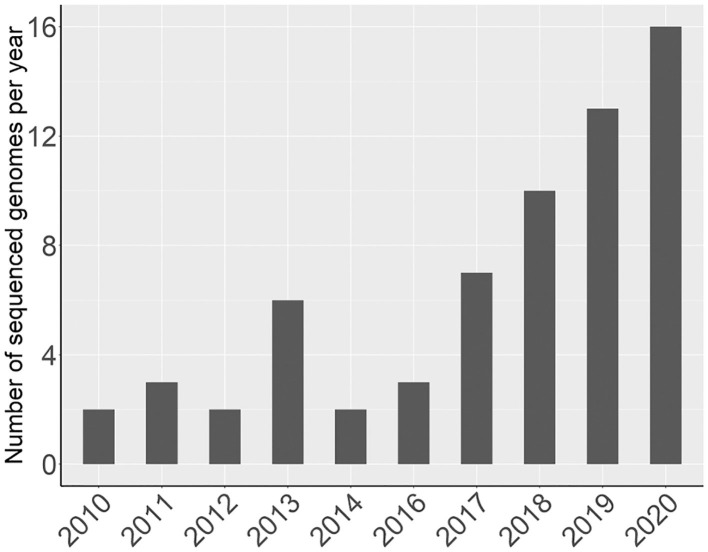
A graph showing the number of newly sequenced Rosaceae genomes per year.

**Figure 2:**
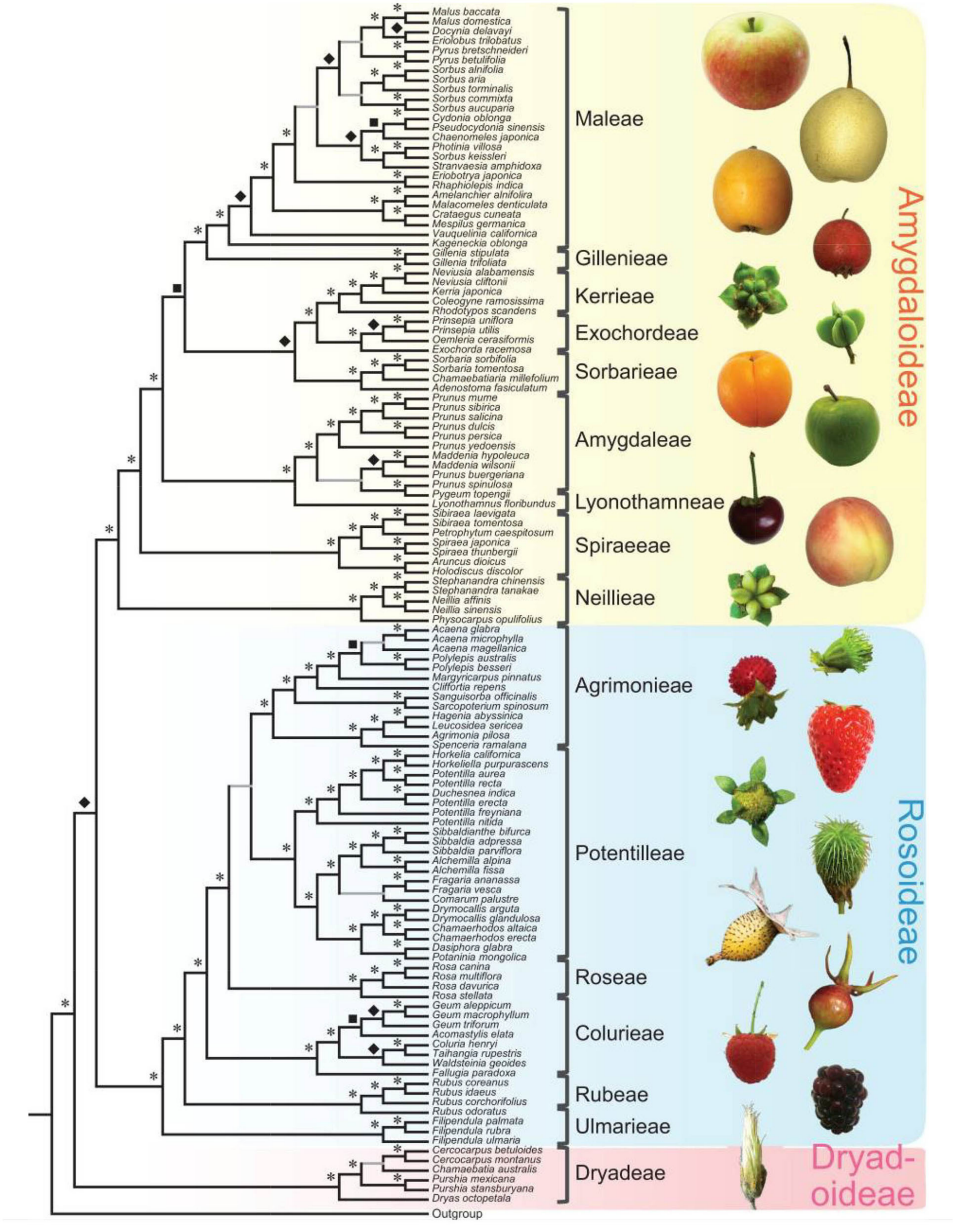
Rosaceae phylogeny and Rosaceae fruit types. At left is the nuclear phylogeny established in Xiang et al. (2017). Asterisks, diamonds, and squares indicate 100, 90, and 80 supports, respectively. Plant photographs on the right show the diversity of Rosaceae fruits. The figure is from Xiang et al. (2017) under the terms of Creative Commons Attribution License (CC BY 4.0).

Since 2016, there has been a rapid increase in the number of new Rosaceae genomes, from three new genomes in 2016 to 16 new genomes in 2020 (Figure 1). This trend will likely accelerate as research groups are moving into pan-genome sequencing. Figure 2 shows the nuclear phylogeny of Rosaceae and illustrates genera with different fruit types. Table 1 summarizes the status of genome sequencing in a selective number of economically important Rosaceae lineages. A more comprehensive summary of Rosaceae genomes and transcriptomes is provided in Supplementary Table 1, in which a total of 72 Rosaceae genomes or transcriptome assemblies are included. In addition, Supplementary Table 1 provides specific information on species name, variety name, ploidy level, and genome assembly as well as annotation versions, references, available websites, associated transcriptomes, and accession numbers for accessing these resources. In the following sections, we discuss and highlight some of the important Rosaceae genome studies.

**Table 1:** List of selective Rosaceae species and corresponding genome resources.

**Species**	**Ploidy**	**Common name**	**Variety**	**Access genome at**	**Species website**	**Reference**
*Malus domestica*	2*n* = 2*x* = 34	Apple	Golden Delicious doubled haploid (GDDH13)	GDR and NCBI Genome	https://iris.angers.inra.fr/gddh13/	Daccord et al., 2017
*Malus domestica*	2*n* = 2*x* = 34	Apple	Hanfu anther-derived homozygous line HFTH1	GDR and NCBI Genome		Zhang et al., 2019
*Pyrus bretschneideri*	2*n* = 2*x* = 34	Chinese white pear	.	GDR	http://genedenovoweb.ticp.net:81/pear/	Xue et al., 2018
*Pyrus communis*	2*n* = 2*x* = 34	European pear	Bartlett doubled haploid	GDR		Linsmith et al., 2019
*Pyrus ussuriensis* x *communis*	2*n* = 2*x* = 34	Harbin x European pear	Zhongai 1	GDR and NCBI Genome		Ou et al., 2019
*Prunus armeniaca*	2*n* = 2*x* = 16	Apricot	Chuanzhihong	GDR		Jiang et al., 2019
*Prunus armeniaca*	2*n* = 2*x* = 16	Apricot	Rojo Pasion	NCBI Genome		Campoy et al., 2020
*Prunus avium*	2*n* = 2*x* = 16	Sweet cherry	Satonishiki	GDR and NCBI Genome	http://cherry.kazusa.or.jp/	Shirasawa et al., 2017
*Prunus avium*	2*n* = 2*x* = 16	Sweet cherry	Big Star	NCBI Genome		Pinosio et al., 2020
*Prunus avium*	2*n* = 2*x* = 16	Sweet cherry	Tieton	NCBI Genome		Wang et al., 2020a
*Prunus avium*	2*n* = 2*x* = 16	Sweet cherry	Tieton	GDR and NCBI Genome		Wang et al., 2020b
*Prunus dulcis*	2*n* = 2x = 16	Almond	Lauranne	GDR and NCBI Genome		Sánchez-Pérez et al., 2019
*Prunus dulcis*	2*n* = 2*x* = 16	Almond	Texas	GDR and NCBI Genome		Alioto et al., 2020
*Prunus mume*	2*n* = 2*x* = 16	Japanese apricot	.	NCBI Genome		Zhang et al., 2012
*Prunus persica*	2n = 2*x* = 16	Peach	Lovell doubled haploid	GDR and NCBI Genome	http://services.appliedgenomics.org/projects/prunus_persica_v2/prunus_persica_v2/intro/index.html	Verde et al., 2017
*Prunus salicina*	2*n* = 2*x* = 16	Japanese plum	Sanyueli	GDR and NCBI Genome		Liu C. et al., 2020
*Prunus yedoensis* var. *nudiflora*	2*n* = 2*x* = 16	King cherry	.	GDR and NCBI Genome		Baek et al., 2018
*Prunus yedoensis*	2*n* = 2*x* = 16	Yoshino cherry	Somei-Yoshino	GDR and NCBI Genome	http://cherry.kazusa.or.jp/	Shirasawa et al., 2019
*Fragaria* x *ananassa*	2*n* = 8*x* = 56	Garden strawberry	Reikou	GDR and NCBI Genome	http://strawberry-garden.kazusa.or.jp	Hirakawa et al., 2014
*Fragaria* x *ananassa*	2*n* = 8*x* = 56	Garden strawberry	Camarosa	GDR		Edger et al., 2019
*Fragaria iinumae*	2*n* = 2*x* = 14	.	.	GDR and NCBI Genome	http://strawberry-garden.kazusa.or.jp	Hirakawa et al., 2014
*Fragaria iinumae*	2*n* = 2*x* = 14	.	.	GDR and NCBI Genome		Edger et al., 2020
*Fragaria vesca*	2*n* = 2*x* = 14	Woodland strawberry	Hawaii 4	GDR		Edger et al., 2018
*Fragaria viridis*	2*n* = 2*x* = 14	.	.	GDR		Feng et al., 2021
*Rosa chinensis*	2*n* = 2*x* = 14	Chinese rose	Old Blush homozygous rose line	GDR and NCBI Genome	https://lipm-browsers.toulouse.inra.fr/pub/RchiOBHm-V2/	Raymond et al., 2018
*Rosa chinensis*	2*n* = 2*x* = 14	Chinese rose	Old Blush doubled haploid	GDR	https://iris.angers.inra.fr/obh/	Hibrand Saint-Oyant et al., 2018
*Rosa multiflora*	2*n* = 2*x* = 14	Multiflora rose	.	GDR and NCBI Genome	http://rosa.kazusa.or.jp	Nakamura et al., 2018
*Rubus idaeus*	2*n* = 2*x* = 14	Red raspberry	Joan J.			Wight et al., 2019
*Rubus occidentalis*	2*n* = 2*x* = 14	Black raspberry	ORUS 4115-3	GDR		VanBuren et al., 2018

### Ornamentals

Two high-quality genomes of Chinese rose (*Rosa chinensis* cv. “Old Blush”) were generated from double haploid or homozygous lines (Hibrand Saint-Oyant et al., 2018; Raymond et al., 2018). The genome assembly by Raymond et al. (2018) consists of 82 contigs with an N50-value of 24 Mb, 36,377 protein-coding genes, and 3,971 long non-coding RNAs (lncRNAs), and the genome by Hibrand Saint-Oyant et al. (2018) is composed of 564 contigs (N50: 3.4 Mb), 39,669 predicted protein-coding genes, and 4,812 non-coding genes. The rose genomes show extensive synteny with the genome of diploid strawberry *Fragaria vesca* and provide valuable resources for identifying the molecular basis of key ornamental traits. For example, the “double flower” rose is more attractive due to large numbers of petals. Taking advantage of the sequenced genome, a GWAS study was conducted with 96 cultivated roses, which led to the identification of a transposon insertion in the intron of an *APETALA2(AP2)/TOE* homolog (Hibrand Saint-Oyant et al., 2018). Hence, the mis-regulated *AP2/TOE* appears to have resulted in reduced expression of *AGAMOUS*, leading to the double-flower phenotype.

**Figure 3:**
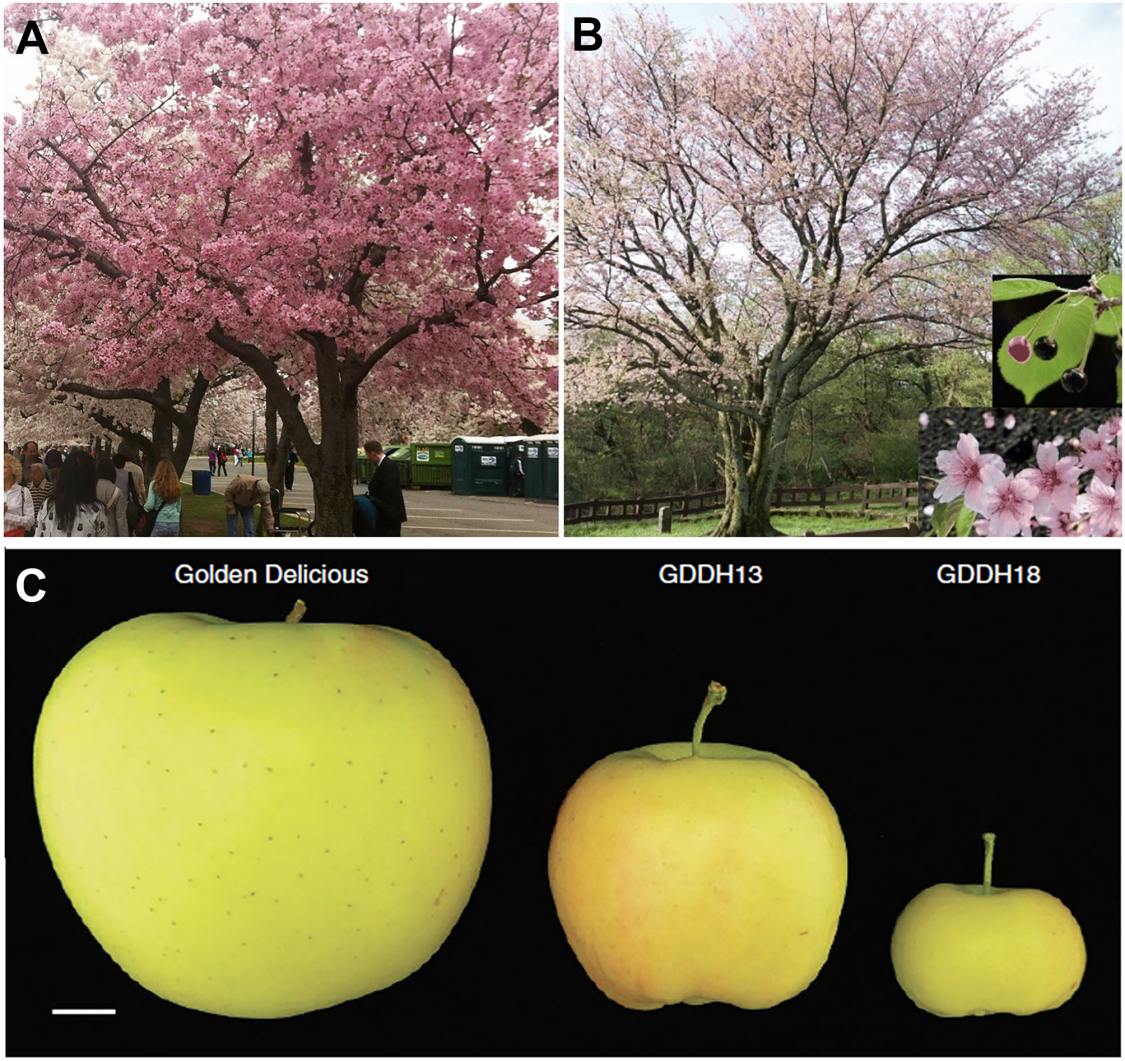
Genomic studies to address questions about genetic relationship in cherry tree and fruit size in apple. **(A)** Yoshino cherry tree (*P. x yedoensis*) in the Washington, D.C., tidal basin. **(B)** A King cherry (*P. yedoensis var. nudiflora* Koehne) has superior flower, fruit, and tree shape. **(C)** A Golden Delicious apple and medium and small size apples from two isogenic lines, GDD13 and GDDH18, derived from the same haploid. The dramatic fruit size difference between GDDH13 and GDDH18 results from epigenetic differences. Photos in **(B)** and **(C)** are from Baek et al. (2018) and Daccord et al. (2017), respectively (both licensed under Creative Commons Attribution License 4.0).

Another worldwide ornamental tree is the flowering cherry native to Korea, Japan, and China. Due to a long history of cultivation, hybridization, and selection, there is confusion concerning the names and origins of many varieties. For example, the relationship between the King cherry (*Prunus yedoensis* var. *nudiflora*), a Korean cherry tree originating on Jeju Island, and the Yoshino cherry (*Prunus x yedoensis*), a popular hybrid cherry tree enjoyed in Japan and other regions of the world (Figures 3A,B), was unknown. A draft genome of King cherry was constructed, and genome-wide variome analysis using the King cherry assembly as a reference revealed that the King and Yoshino cherry trees can be clearly distinguished genetically (Baek et al., 2018).

### Pome Fruits

Pear and apple share a recent whole-genome duplication event that occurred prior to their divergence and may underlie their pome fruit type (Xiang et al., 2017; Li et al., 2019). Pome fruits are characterized by their hypanthium-derived fruit flesh and agronomic importance world-wide. Multiple species and varieties of apple have been sequenced, including *Malus domestica* Golden Delicious (Daccord et al., 2017), *Malus domestica* Hanfu (Zhang et al., 2019), and *Malus baccata* (Chen et al., 2019) (Supplementary Table 1). Similarly, multiple species of pear, such as Chinese white pear *Pyrus bretschneideri* (Wu et al., 2013; Xue et al., 2018), European pear *Pyrus communis* “Bartlett” (Chagné et al., 2014; Linsmith et al., 2019), *Pyrus ussuriensis x communis* (Ou et al., 2019), and a wild Birchleaf pear (*Pyrus betulifolia*-Shanxi Duli, *Pbe-*SD) (Dong et al., 2020), have been sequenced (Supplementary Table 1). The ability to generate a double haploid line of “Golden Delicious” (GDDH13) provides an advantage in genome assembly (Daccord et al., 2017). Sequence analysis shows a major burst of different transposable elements (TEs) around 21 million years ago in the precursor of modern apple. The authors propose that the TE bursts may have possibly contributed to the divergence of apple from pear (Daccord et al., 2017). In addition, the higher quality genome allows the exploration of epigenomes and epigenetic effects on agronomic traits, such as fruit size (Figure 3C). GDDH13 and GDDH18 are isogenic lines obtained from the same haploid, but they develop different sized fruit. Whole genome bisulfide sequencing identified 22 genes with differentially methylated regions in their promoters at two developmental stages, three of which, SPL13 (MD16G0108400), ACS8 (MD15G0127800), and CYP71A25 (MD14G0147300), possess increased methylation in GDDH18 and could potentially contribute to the smaller fruit size of GDDH18 (Daccord et al., 2017).

### Drupe, Stone Fruit, and Sweet Almond

*Prunus* develops drupe fruit, typically botanic fruit with ovary wall-derived fruit flesh (Figure 2). They include many agronomically important species, including peach, apricot, sweet cherry, and plum. They are also called stone fruit because their seeds are enclosed by large and hard (stony) endocarps. Almond (*Prunus dulcis*) is a closely related species with a highly syntenic genome to these *Prunus* species (Dirlewanger et al., 2004), but it offers high-value seeds instead of fleshy fruits. The genome resemblance among the Prunus species explains why they can form inter-specific hybrids. Although wild almonds accumulate a bitter and toxic amygdalin in their seeds, domesticated almonds develop sweet kernels/seeds. The genetic basis of this important domestication trait was recently revealed when the almond genomes were sequenced by two research groups using almond cultivars, Lauranne and Texas, respectively (Sánchez-Pérez et al., 2019; Alioto et al., 2020). The two genomes have similar contig N50-values (Lauranne: 82.26 kb, Texas: 103 kb) and protein-coding genes (Lauranne: 27,817, Texas: 27,969). Subsequent mapping identified a point mutation (Leu to Phe) in the bHLH2 gene that normally controls the expression of two P450 monooxygenase genes *CYP79D16* and *CYP71AN24* required for amygdalin biosynthesis (Sánchez-Pérez et al., 2019). The mutant bHLH2 fails to form a functional dimer, resulting in loss of P450 gene expression and, hence, sweet kernels. Alioto et al. (2020) compare the genomes between *Prunus dulcis* cv. Texas and peach, sweet cherry, and Japanese apricot and observed highly methylated TE insertions surrounding the *CYP71AN24* gene, whose reduced expression was correlated with the sweet kernel trait in the almond cultivar Texas. Therefore, natural mutations and transposable elements contribute to the diversification of *Prunus* species and domestications of almond.

Following the publication of the peach (*Prunus persica*) genome and subsequent improvement (Verde et al., 2013, 2017), the pan-genome of peach (*P. persica*) is a much-welcomed next step (Cao et al., 2020). A pan-genome consists of the entire set of genes and genetic variations within a species, and the portion of the pan-genome common to all cultivars in the species forms the core genome. A pan-genome identifies genetic variations among cultivars, provides valuable resources, and supports evolutionary studies. In this study, 100 *P. persica* accessions were sequenced, giving rise to 27,796 genes in the pan-genome. Furthermore, the genomes of four wild peaches (*Prunus mira, Prunus kansuensis, Prunus davidiana, Prunus ferganensis*) were assembled *de novo*, and the core genome shared by peach and its four wild relatives consists of 15,216 gene families. The analysis reveals dramatic variation in gene content between congeneric species and suggests that *P. mira* is the primitive ancestor of the cultivated peach.

## New Technologies for Improving Genome Assembly and Annotation

The rapid development of sequencing and related technologies, such as PacBio single-molecule real-time (SMRT) sequencing, Oxford Nanopore sequencing, Hi-C, and BioNano optical mapping over the past 10 years have greatly facilitated genome assembly and annotation. PacBio and Nanopore both belong to the third-generation (single-molecule and real-time) sequencing technology. Their long-read DNA-seq helps overcome challenges of genome assembly caused by repetitive regions (Rhoads and Au, 2015; Lu et al., 2016; Jiao and Schneeberger, 2017) and facilitates splicing isoform prediction and genome annotation (Rhoads and Au, 2015). Hi-C and BioNano optical mapping are two scaffolding technologies that help to construct chromosome-level scaffolds from contigs by providing long-range genomic information (Korbel and Lee, 2013; Tang et al., 2015; Jiao and Schneeberger, 2017). Many important crop species' genomes have benefitted from several rounds of genome assembly and annotation whenever a new technology was applied.

For heterozygous diploid species, most genomes were assembled into one pseudo-haploid sequence, ignoring sequence or structural differences between the two parental chromosomes. To generate homozygous lines, traditional methods involve breeding or creating double haploids; however, this can be extremely time-consuming or technically challenging. A recent advancement involves single-cell sequencing of haploid gametes, which enables separation of whole genome sequencing reads into haplotype-specific read sets. Using this method, two haploid genomes of a diploid apricot tree (*Prunus armeniaca* cultivar “Rojo Pasion”) were assembled based on whole genome sequencing of 445 pollen grains (Campoy et al., 2020). This is a much-needed advancement applicable to other Rosaceae species.

### Several Updates in Apple Genome Assembly and Annotation

The progressive improvement of apple genome assemblies nicely illustrates the application of newer technologies. The first genome of apple (*Malus domestica* cv. “Golden Delicious”) was published in 2010 using traditional Sanger sequencing and 454 next-generation sequencing (Velasco et al., 2010). Six years later, an improved apple genome of “Golden Delicious” was assembled based on a combination of Illumina short reads and PacBio long reads (Li et al., 2016). Accordingly, the contig N50 of the apple genome was 111,619 bp, almost seven times the previous N50 (16,171 bp). In 2017, another *de novo* genome assembly of double haploid “Golden Delicious” (GDDH13) was published (Daccord et al., 2017). In addition to the Illumina and PacBio data, a BioNano optical mapping was used in scaffolding. As a result, the scaffold N50 was increased to 5,558 kb. In 2019, Illumina, PacBio, BioNano, and Hi-C technologies were integrated to construct a high-quality genome assembly of “Hanfu” (HFTH1) apple, a *Malus domestica* cultivar grown in northern China (Zhang et al., 2019). The scaffold N50 was increased to 6,988 kb. Compared with the HFTH1 genome, the three published “Golden Delicious” genomes shared 11,502 deletions and 6,590 insertions with an average length of 508 bp and 519 bp, respectively (Velasco et al., 2010; Li et al., 2016; Daccord et al., 2017). The average density of shared SNPs with the “Golden Delicious” genomes is 2.15/kb. The HFTH1 genome was utilized to completely fill 488 gaps in the GDDH13 genome; the average length of the filled gaps is 78,864 bp (Zhang et al., 2019). It would be useful if the gap-filled GDDH13 genome could be made publicly available. Because of the genetic variations between “Hanfu” and “Golden Delicious,” it is beneficial to use the genome assembly of the cultivar most closely related to the cultivars under one's study as a reference.

In addition to genome assembly, high-quality genome annotations are essential to enhance the utility of the genome. In the first “Golden Delicious” genome published in 2010, the genome annotation was based on the gene prediction programs and ESTs from Genbank (Korf et al., 2001; Birney et al., 2004; Majoros et al., 2004; Solovyev et al., 2006; Velasco et al., 2010; Sayers et al., 2020). In 2014, an improved apple reference transcriptome was constructed using RNA-Seq data generated from “Golden Delicious” fruits at 14 time points during development (Bai et al., 2014). In 2016, the *de novo* “Golden Delicious” genome assembly was supplemented by annotations based on RNA-Seq data from three distinct tissues (leaves, flowers, and stems) as well as *ab initio* and protein homology-based predictions (Li et al., 2016). To annotate the latest “Golden Delicious” GDDH13 genome, mRNA was extracted and sequenced from more tissues, including leaves, roots, fruits, apex, stems, and flowers (Daccord et al., 2017). The GDDH13 genome annotation has the lowest number of protein-coding genes at 42,140 (Daccord et al., 2017) compared with 53,922 (Li et al., 2016) and 63,141 (Velasco et al., 2010). However, GDDH13 possesses the highest BUSCO completeness at 94.9% (Daccord et al., 2017) compared with 51.5% (Li et al., 2016) and 86.7% (Velasco et al., 2010).

### Several Updates in Strawberry Genome Assembly and Annotation

As with apples, the diploid woodland strawberry (*Fragaria vesca* ssp. *vesca* “Hawaii4”) genome assembly and annotation went through several rounds of updates. The first woodland strawberry genome became available at the end of 2010, and its genome annotation (v1.1) was generated by GeneMark-ES+ (Lomsadze et al., 2005), which integrated *ab initio* gene prediction and EST evidence (Shulaev et al., 2011). In 2015, a new annotation (v1.1.a2) was created that combined different evidence, such as *de novo* and genome-guided transcriptome assembly from RNA-Seq reads, *ab initio* gene models, and plant protein sequences from UniProt (Darwish et al., 2015). More than 2000 new genes were added in the v1.1.a2 annotation. In 2014, dense linkage maps were leveraged to construct an improved woodland strawberry genome assembly (v2.0.a1) (Tennessen et al., 2014). In 2017, based on PacBio long reads and Illumina short reads from *F. vesca* fruit receptacles as well as prior short-read RNA-Seq data, a new annotation (v2.0.a2) was generated (Li et al., 2018). Although the total number of protein-coding genes decreased slightly, 13,168 protein-coding genes were updated in their gene structures, alternatively spliced (AS) isoforms were identified for 7,370 genes, and the BUSCO completeness score was increased to 95.7% from the prior version (88.9%).

At the end of 2017, a high-quality woodland strawberry genome (v4.0.a1) was assembled using PacBio long reads, Illumina short reads, and BioNano optical mapping (Edger et al., 2018). This version uses a different gene-naming system, moving from the geneXXXXX to FvH4XgXXXXX format. Li Y. et al. (2019) include a supplementary table in their publication that correlates the *F. vesca* gene names between the old and new naming systems. In addition, a new annotation (v4.0.a2) was created based on comprehensive short- and long-read RNA-Seq data (Li Y. et al., 2019), adding 5,419 new protein-coding genes, improving the BUSCO completeness score to 98.1% from the prior 91.1%, and adding AS isoforms detected for about 30% of the genes.

In 2013, the first draft octoploid garden strawberry genome (*Fragaria x ananassa* cv. “Reikou”) was reported (Hirakawa et al., 2014). Homoeologous sequences of the allo-octoploid strawberry are integrated into a haploid genome named FANhybrid_r1.2 with an N50 of 5.14 kb. Gene prediction was done *ab initio* using Augustus. In the same study, the genomes of several Fragaria species were sequenced and assembled, including *F. orientalis, F. iinume, F. nipponica*, and *F. bucharica*, and *F. bucharica* (USDA accession CFRA522) was originally misidentified as *Fragaria nubicola* (Tennessen et al., 2014). In 2019, a near-complete chromosome-scale assembly of the *Fragaria x ananassa* cv. “Camarosa” was constructed with a contig N50 of about 79.97 kb, taking advantage of Illumina, 10X Genomics, and PacBio long reads (Edger et al., 2019). This chromosome-scaled genome consists of A, B, C, and D subgenomes, and the genome annotation (v1.0.a1) utilized RNA-Seq data from diverse tissue types (108,087 protein-coding genes) (Edger et al., 2019). In the same year, a garden strawberry reference transcriptome was constructed using PacBio sequencing (Yuan et al., 2019). The PacBio data in this study, together with other publicly available Illumina RNA-Seq data were recently utilized to improve the annotation of the *Fragaria x ananassa* cv. “Camarosa” genome (v1.0.a1) (Liu et al., 2021). Compared with *Fragaria x ananassa* v1.0.a1, the new annotation v1.0.a2 had a slight increase in the number of protein-coding genes (108,447). Importantly, the new annotation (v1.0.a2) for *Fragaria x ananassa* cv. “Camarosa” includes AS isoforms for 11,044 genes and adds 5′ and 3′ UTR information to a large proportion of the protein-coding genes (v1.0.a1: 38.93%, v1.0.a2: 73.61%).

The complete genome sequencing of the *Fragaria x ananassa* cultivar “Camarosa” allowed the identification of diploid progenitors, which has long been a mystery and recently a topic of intense debate. Based on the tree-searching algorithm (PhyDS), Edger et al. (2019) propose four diploid species (*F. vesca, F. iinumae, F. viridis*, and *F. nipponica*) as the four progenitors of the octoploid and suggest the hexaploid *F. moschata* as an intermediate species (Edger et al., 2019). However, Liston et al. (2020) reanalyzed the four subgenomes in a phylogenomic context and found support for *F. vesca* and *F. iinumae* but disputed *F. viridis, F. nipponica*, and *F. moschata* as progenitors (Liston et al., 2020). In response, a new chromosome-scale genome of *F. iinumae* was subsequently assembled, and a reanalysis using PhyDS supports their original proposal regarding the four diploid species as the progenitors (Edger et al., 2020). A third group recently sequenced and assembled the genomes of three wild diploid species, *F. nilgerrensis, F. nubicola*, and *F. viridis* (Feng et al., 2021). Combining these three genomes with the previously sequenced *F. vesca* and *F. iinumae* genomes, the group utilized sppIDer (Langdon et al., 2018) to map short-read sequencing data of *F. x ananassa* to a composite reference genome, and the result supports that *F. vesca* and *F. iinumae*, but not others, are the progenitor species of the cultivated garden strawberry (Feng et al., 2021).

## Non-coding RNA in Rosaceae Genomes

Non-coding RNAs (ncRNAs) are RNAs that do not encode proteins. They are important components of the genomes and play roles in plant development and stress responses (Liu et al., 2017). However, ncRNA prediction is not always included in the annotation of published genomes. Computational tools, such as tRNAscan-SE (Chan and Lowe, 2019) and RNAmmer (Lagesen et al., 2007) are used to predict tRNA and rRNAs, respectively. Infernal (Nawrocki and Eddy, 2013) and Rfam (Kalvari et al., 2018) are often used to identify different types of ncRNAs. Besides the commonly used tools mentioned, additional approaches can be applied to detect ncRNAs, especially small RNAs. To predict miRNAs in the apricot (*Prunus armeniaca* cv. “Chuanzhihong”) genome, miRNA sequences derived from miRbase (Kozomara et al., 2019) were mapped against the genome, and the resulting miRNA candidates were further verified by RNAfold analysis (Lorenz et al., 2011; Jiang et al., 2019). In *Rosa chinensis* cv. “Old Blush,” an RNA library from pooled tissues was sequenced and analyzed for miRNA identification; tRNA and rRNA-like sequences were removed first, and miRNA precursors were then annotated using an established pipeline (Formey et al., 2014; Raymond et al., 2018). Previously, small RNA libraries derived from diverse tissues were sequenced to detect miRNAs and PhasiRNAs in wild diploid strawberry using established pipelines and criteria (Meyers et al., 2008; Xia et al., 2012, 2015). The same small RNA sequencing data sets were later used to identify small RNAs during the woodland strawberry genome reannotations (v2.0.a2 and v4.0.a2) (Axtell, 2013; Li et al., 2018; Li Y. et al., 2019). In addition to small RNAs, lncRNAs, a class of ncRNAs with length >200 bp, are shown to possess epigenetic regulatory roles in key cellular processes. RNA-Seq data from woodland strawberry flower and fruit tissues were used to identify lncRNAs, leading to 5,884 lncRNAs (Kang and Liu, 2015). In 2017, in updating woodland strawberry genome annotation v2.0.a2, an updated prediction of 4,042 lncRNA was included (Li et al., 2018).

## Computational Databases for Rosaceae Species

Computational databases are becoming indispensable tools for research. Below, we discuss databases, highlighting those that are of particular importance to Rosaceae research. Although Table 1 and Supplementary Table 1 provide species-specific websites that accompany the genome-sequencing papers, Table 2 lists highly useful databases with various analysis tools and information.

**Table 2:** List of websites/databases useful for Rosaceae research.

**Database name**	**URL**	**Species**	**Information provided**	**Reference**
**ROSACEAE DATABASES**				
GDR (Genome Database for Rosaceae)	https://www.rosaceae.org	Rosaceae	Genomic	Jung et al., 2019
MDR (Methylation Database for Rosaceae)	http://mdr.xieslab.org	Rosaceae	Methylation	Liu et al., 2019
AppleMDO	http://bioinformatics.cau.edu.cn/AppleMDO	*Malus domestica*	Co-expression	Da et al., 2019
*Fragaria vesca* co-expression network explorer	http://159.203.72.198:3838/fvesca	*Fragaria vesca*	Co-expression	Shahan et al., 2018
SGR (Strawberry Genome Resources)	http://bioinformatics.towson.edu/strawberry	*Fragaria vesca*	Transcriptome	Darwish et al., 2013
SGD (Strawberry Genome Database)	*http://www.strawberryblast.ml:8080/strawberry/viroblast.php*	*Fragaria x ananassa*	Genomic	Liu et al., 2021
TRANSNAP	http://plantomics.mind.meiji.ac.jp/nashi	*Pyrus pyrifolia*	Transcriptome	Koshimizu et al., 2019
**GENERAL PLANT DATABASES**				
NCBI Genome	https://www.ncbi.nlm.nih.gov/genome	General	Genomic	Tatusova et al., 1999
PLAZA	https://bioinformatics.psb.ugent.be/plaza	Plant	Genomic	Van Bel et al., 2018
Phytozome	https://phytozome-next.jgi.doe.gov	Plant	Genomic	Goodstein et al., 2012
EnsemblPlants	https://plants.ensembl.org/index.html	Plant	Genomic	Bolser et al., 2017
PMN (Plant Metabolic Network)	https://plantcyc.org	Plant	Functional (Pathway)	Schläpfer et al., 2017
Plant Reactome	https://plantreactome.gramene.org/index.php?lang=en	Plant	Functional (Pathway)	Naithani et al., 2020
PlantTFDB (Transcription Factor Database)	http://planttfdb.gao-lab.org	Plant	Functional (TF Regulation)	Jin et al., 2017
PlantRegMap (Plant Transcriptional Regulatory Map)	http://plantregmap.gao-lab.org	Plant	Functional (TF Regulation)	Tian et al., 2020
Next-Gen Sequence Databases and sRNA Tools	https://mpss.danforthcenter.org	Plant	ncRNA (small RNA)	Nakano et al., 2020
CANTATAdb	http://cantata.amu.edu.pl	Plant	ncRNA (lncRNA)	Szcześniak et al., 2016
RPTEdb (Rosaceae Plant Transposable Element Database)	http://genedenovoweb.ticp.net:81/RPTEdb/index.php	Rosaceae	TE	Ma et al., 2018
PlantRGDB (Plant Retrocopied Gene DataBase)	https://probes.pw.usda.gov/plantrgdb/index.php	Plant	Retrocopied Gene	Wang, 2017

### Rosaceae Genome Databases

Genome Database for Rosaceae (GDR) (www.rosaceae.org) (Jung et al., 2019) is, by far, the best resource hub for Rosaceae research. It hosts the most comprehensive and up-to-date collection of genome assembly and annotation versions for widely studied genera, *Fragaria, Malus, Prunus, Potentilla, Pyrus, Rosa*, and *Rubus*. For instance, GDR hosts *Fragaria vesca* genome assemblies of v1.0, v1.1 (an improved pseudochromosome assembly of v1.0), v2.0.a1, and v4.0.a1. Moreover, it incorporates corresponding updated annotations v1.1.a2, v2.0.a2, and v4.0.a2. In addition, GDR serves as the database of record for Rosaceae gene names; standardized gene-naming guideline should be followed to ensure uniformity and clarity (Jung et al., 2015). Besides the genes and genomes, GDR provides genetic maps, markers, germplasm, and trait information as well as an impressive set of tools. For example, the search tools of GDR enable users to search for specific gene sequence, maps, and markers; its MegaSearch tool allows downloading different data types in bulk. With the GDRCyc tool, users can search, visualize, and overlay pathway data. With the Synteny Viewer tool, one can select specific Rosaceae species for comparison, visualize syntenic blocks, and obtain information on syntenic genes.

The NCBI Genome (https://www.ncbi.nlm.nih.gov/genome) (Tatusova et al., 1999) on the other hand collects genomes from a broader range of Rosaceae species (Supplementary Table 1), including lesser-known species, such as Drummond's mountain avens (*Dryas drummondii*), wood avens (*Geum urbanum*), and bitterbrush (*Purshia tridentate*) (Griesmann et al., 2018; Jordan et al., 2018).

### Rosaceae Species-Specific Databases

Many genome sequencing or annotation papers of Rosaceae species are accompanied by species-specific websites that provide tools, including BLAST searches for genes of interest. The URLs for these websites are included in Table 1 (or Supplementary Table 1 with a complete list). For instance, the genomes of Yoshino cherry (*Cerasus* x *yedoensis*) and sweet cherry (*Prunus avium*) are both deposited in DBcherry (http://cherry.kazusa.or.jp/) (Shirasawa et al., 2017, 2019). The built-in BLAST enables users to search their sequences of interest against the cherry genomes, and JBrowse is embedded in the database for visualizing the genomic regions. The genomes of garden strawberry (*Fragaria x ananassa*) and multiflora rose (*Rosa multiflora*) are available in Strawberry GARDEN (http://strawberry-garden.kazusa.or.jp/) and Rosa multiflora DB (http://rosa.kazusa.or.jp/), respectively (Hirakawa et al., 2014; Nakamura et al., 2018). These two websites as well as the database for cherry are all supported by the Kazusa DNA Research Institute.

Several Rosaceae species have developed species-specific databases with multiple analysis tools and resources, which are summarized in Table 2 and described below. Strawberry Genomic Resources (SGR, http://bioinformatics.towson.edu/strawberry/default.aspx) is a website that integrates different types of woodland strawberry (*Fragaria vesca*) genomic data (Darwish et al., 2013). It allows users to access the transcriptome analysis of the woodland strawberry early fruit development (Kang et al., 2013). Users can acquire differentially expressed genes between distinct tissues and stages by searching the database and use the eFP browser to visualize RNA-Seq data across tissues and stages for genes of interests (Hawkins et al., 2017). An updated *F. vesca* eFP browser is hosted at the ePlant (http://bar.utoronto.ca/). In addition, a recent annotation update of the *Fragaria x ananassa* cv. “Camarosa” genome (v1.0.a2) is accompanied with a separate website, “Strawberry Genome Database” (Table 2), that allows users to search for garden strawberry genes (Liu et al., 2021).

A reference transcriptome of Chinese pear (*Pyrus pyrifolia*) was constructed by utilizing PacBio, 454, and Sanger sequencing, and it is stored in the database TRANSNAP (http://plantomics.mind.meiji.ac.jp/nashi/) (Koshimizu et al., 2019). The database also includes gene functional annotation performed by BLASTP (Altschul et al., 1990), KAAS (Moriya et al., 2007), and InterProScan (Jones et al., 2014). Users can examine gene-expression patterns generated from GEO (https://www.ncbi.nlm.nih.gov/geo/) microarray data.

The *Fragaria vesca* gene co-expression network explorer (http://159.203.72.198:3838/fvesca/) was developed to host the non-consensus and consensus co-expression networks generated using RNA-Seq data from flower and fruit tissues of the woodland strawberry (Shahan et al., 2018). Users are able to search for genes of interest and the transcriptional co-expression clusters to which they belong, obtain network statistics, visualize cluster eigengene expression, examine enriched GO terms in the cluster of interest, and download the cluster graphml structure.

AppleMDO (http://bioinformatics.cau.edu.cn/AppleMDO/) is a multidimensional omics database for apple co-expression networks and chromatin states (Da et al., 2019). The global co-expression network was constructed using transcriptomes from a variety of tissues, stages, and stress treatments. The tissue-preferential network was built based on RNA-Seq data sets of different tissues without stress treatments. A combination of ChIP-seq, DNase-seq, and BS-seq data sets were utilized by ChromHMM (Ernst and Kellis, 2012) to predict the chromatin states. Furthermore, AppleMDO offers tools to perform GO analysis and motif scan.

Methylation Database for Rosaceae (http://mdr.xieslab.org/) is a database presenting methylation analyses of Rosaceae species, including woodland strawberry and Chinese rose (*Rosa chinensis*) (Liu et al., 2019). Using PacBio sequencing data that is publicly available (Edgar et al., 2018; Raymond et al., 2018), DNA N6-methyladenine and N4-methylcytosine modifications were identified for woodland strawberry and Chinese rose with the PacBio SMRT analysis software.

The Rosaceae Plant TE Database (RPTEdb, http://genedenovoweb.ticp.net:81/RPTEdb/index.php) provides information on TEs in five Rosaceae species: woodland strawberry, apple, Japanese apricot (*Prunus mume*), Chinese white pear, and peach (Ma et al., 2018). The TEs were detected in three ways, *de novo* identification performed by PILER (Edgar and Myers, 2005) and RepeatModeler (http://www.repeatmasker.org/), signature-based identification achieved by LTR_STRUC (McCarthy and McDonald, 2003) and LTR_FINDER (Xu and Wang, 2007), and similarity-based identification conducted by RepeatMasker (http://www.repeatmasker.org/) using the Repbase database (Jurka et al., 2005; Bao et al., 2015). Users can search and download TEs in each TE family or superfamily and view TE trees constructed using a superfamily of TEs from five Rosaceae species.

### Useful Plant Databases for Comparative Genomics, Metabolic Networks, and Others

Although the summary above focuses on Rosaceae databases, many plant databases are also highly useful for Rosaceae research. Table 2 lists some of the most useful ones, such as Plant Transcription Factor Database (http://planttfdb.gao-lab.org/), Plant Transcriptional Regulatory Map (http://plantregmap.gao-lab.org/), CANTATAdb (http://cantata.amu.edu.pl/) for plant lncRNAs, and Plant Retrocopied Gene DataBase (http://probes.pw.usda.gov/plantrgdb) for plant retrocopied genes (Wang, 2017). Below, we highlight four such databases.

PLAZA (https://bioinformatics.psb.ugent.be/plaza/) (Van Bel et al., 2018) and Phytozome (https://phytozome-next.jgi.doe.gov/) (Goodstein et al., 2012) are databases for plant genome comparisons. Currently, Dicots PLAZA 4.5 has integrated genomic resources from 55 species, including four Rosaceae species, apple (*Malus domestica*), Chinese white pear (*Pyrus bretschneideri*), peach (*Prunus persica*), and woodland strawberry (*Fragaria vesca*). Phytozome v13 has gathered 224 annotated genomes, including three Rosaceae species, woodland strawberry, apple, and peach. The genome assemblies at PLAZA and Phytozome are not always up to date. For instance, older versions of woodland strawberry genome v1.1 and v2.0.a2 are, respectively, hosted at PLAZA and Phytozome at the moment.

Plant Metabolic Network (PMN, https://plantcyc.org/) (Schläpfer et al., 2017) and Plant Reactome (https://plantreactome.gramene.org/index.php?lang=en) (Naithani et al., 2020) are both databases for plant pathways. Plant Metabolic Network is focused on metabolic pathways and hosts the database PlantCyc that contains shared pathways among more than 350 plant species. Additionally, a single-species database was also constructed in PMN, which allows users to access pathways and enzymes for individual species. PpersicaCyc, SweetcherryCyc, MdomesticaCyc, EuropeanpearCyc, Fvesca_VescaCyc, RmultifloraCyc, and RchinensisCyc are developed for Rosaceae family members. Besides the metabolic pathways, Plant Reactome hosts different types of pathways, including gene regulatory pathways, hormone signaling pathways, and others. Users can view and interact with the pathways in the browser and identify chemical compounds and proteins involved in the processes. The database encompasses multiple Rosaceae species, such as peach, woodland strawberry, and apple. Furthermore, the database enables researchers to perform pathway enrichment analysis and species comparison between pathways of rice and those of selected species.

## Discussion

As a result of revolutionary improvements in DNA sequencing and analysis software, Rosaceae genome research has seen a dramatic jump in the number of sequenced genomes, transcriptomes, databases, and publications. These genomic data and databases will greatly facilitate the understanding of physiology, growth and development, stress responses, adaptation, and domestication of Rosaceae species, laying the foundation for trait improvement through breeding and genome engineering. This view is also shared by a prior review on the genomes of several commercially important Rosaceae plants (Soundararajan et al., 2019). However, there is still ample room for improvement to fully reap the benefit of the genome sequencing revolution. These include increasing the quality and accuracy of Rosaceae genome assemblies and annotations, in particular, for polyploid and hybrid cultivars; identification and incorporation of AS variants and ncRNA into genome annotations; expansion of pan-genome analyses; and establishing robust molecular markers. Development of user-friendly databases that integrate, organize, and coordinate different data types and species is also essential to increase the accessibility and impact of the ever-increasing genomic data sets. The genomic revolution will likely propel significant research progress and further increase the agronomic value of Rosaceae fruits, seeds, and ornamentals.

## Author Contributions

ML and ZL conceived and drafted the manuscript. ML and YX collected information and data. SM provided advice and revised the manuscript. All authors contributed to the article and approved the submitted version.

## Conflict of Interest

The authors declare that the research was conducted in the absence of any commercial or financial relationships that could be construed as a potential conflict of interest.

## Supplementary Material

Supplementary Table 1 A comprehensive list of Rosaceae genomes and relevant information.
